# The Epithelial to Mesenchymal Transition Related Gene Calumenin Is an Adverse Prognostic Factor of Bladder Cancer Correlated With Tumor Microenvironment Remodeling, Gene Mutation, and Ferroptosis

**DOI:** 10.3389/fonc.2021.683951

**Published:** 2021-06-03

**Authors:** YiHeng Du, WenHao Miao, Xiang Jiang, Jin Cao, Bo Wang, Yi Wang, Jiang Yu, XiZhi Wang, HaiTao Liu

**Affiliations:** ^1^ Department of Urology, Suzhou Kowloon Hospital, Shanghai Jiaotong University School of Medicine, Suzhou, China; ^2^ Department of Urology, Shanghai General Hospital, Shanghai Jiaotong University School of Medicine, Shanghai, China; ^3^ Department of Pathology, Suzhou Kowloon Hospital, Shanghai Jiaotong University School of Medicine, Suzhou, China

**Keywords:** bladder cancer, calumenin, tumor microenvironment, immunotherapy, gene mutation, ferroptosis

## Abstract

The tumor microenvironment (TME) plays a critical regulatory role in bladder cancer (BLCA) progression and metastasis. Epithelial-mesenchymal transition (EMT) presents as an essential mechanism of tumor invasion and metastasis. Accumulating pieces of evidence indicated that several microenvironmental factors, including fibroblasts, endothelial, and immune cells, induced EMT in tumor cells. As a hallmark gene of the EMT process, calumenin (CALU) was previously reported to directly impact cancer metastasis. However, the functions and molecular mechanisms of CALU have been rarely reported in BLCA. By multi-omics bioinformatics analysis of 408 TCGA BLCA patients, we demonstrated that CALU was an independent risk factor for BLCA outcome. Subsequently, we verified the correlation of CALU with cancer-associated fibroblasts (CAFs) and tumor-infiltrating immune cells. The results suggested a positive correlation of CALU with CAFs, CD8+ T cells and macrophages. Also, CALU was significantly associated with multiple immune checkpoint-related genes, which ultimately influenced patients’ responsiveness to immunotherapy. Further, we found that the impact of CALU on BLCA prognosis might also be correlated with gene mutations and ferroptosis. Finally, we validated the roles of CALU by single-cell RNA sequencing, PCR and immunohistochemistry. In conclusion, we found that CALU affected BLCA prognosis associated with multiple mechanisms, including TME remodeling, gene mutation and ferroptosis. Further studies on CALU may provide new targets for BLCA immunotherapy and precision medicine.

## Introduction

Urinary bladder cancer (BLCA) is one of the most incident cancers, ranking ninth in prevalence worldwide (1). It is estimated that the morbidity of BLCA will increase in the future because of the increased exposure to BLCA-causing agents and global aging (2). Approximately one-quarter of BLCAs are muscle-invasive bladder cancers (MIBCs), whose incidence and mortality are elevating (3). In 1976, Morales et al. used intravesical Bacillus Calmette-Guérin (BCG) instillation to treat superficial BLCA, making a breakthrough in BLCA treatment (4). However, the prognosis and treatment for MIBC have not made significant progress until the adventure of immunotherapy for BLCA (5). A large number of clinical trials currently employing immunotherapeutic agents are a testament to the tremendous advances they have made in BLCA treatment (6). The effectiveness of immunotherapy in BLCA may be attributed to the large number of immune cells infiltrated within the tumor microenvironment (TME) of BLCA (7, 8).

The TME comprises cellular components such as infiltrated immune cells, stromal cells, cancer cells, and non-cellular components, including extracellular matrix and various types of soluble biological factors or mediators (9). Characterized by sub-regions of nutrient deprivation, low extracellular pH, high interstitial fluid pressure, and hypoxia (10), the TME contributed to genetic instability and further promoted tumor growth. Stromal components of the TME had lots of crosstalks with tumor cells. Such crosstalks could shape the TME into a tumor-promoting one through multiple ways, including Epithelial-mesenchymal transition (EMT), inhibition of ferroptosis and autophagy (11), influencing energy metabolism (12) and tumor infiltrated immune cells (TIICs) (13). EMT presented as an essential mechanism of tumor invasion and metastasis. Stromal cells comprised a crucial source of EMT-related gene expression and further impacted the response to immune checkpoint blockade (ICB) therapy and patients’ survival by altering T cell infiltration in BLCA (14).

Calumenin (CALU), a hallmark gene of the EMT process, was previously reported to directly impact cancer metastasis in multiple cancers. Nagano K et al. reported that CALU was expressed at a significantly higher level in the lung tissues of metastasis-positive cases than in metastasis-negative cases (15). Kunita A et al. demonstrated that CALU was secreted by cancer-associated fibroblasts (CAFs) and increased lung cancer cell proliferation (16). A recent manuscript by Nasri Nasrabadi P et al. also indicated the metastasis promoting role of CALU in colon and lung cancers (17). From the above evidence, we could see the cancer-promoting and CAFs associated roles of CALU. However, the biological behavior that CALU may participate in has not been reported yet in BLCA.

Bioinformatics methods are now widely applied in cancer research. The next-generation RNA sequencing (NGS) and the single-cell RNA (scRNA) sequencing have provided tremendous help in cancer research, especially in TME heterogeneity. In the present study, we conducted a comprehensive study on the mechanisms of CALU in impacting the prognosis of BLCA. With the help of multi-omics bioinformatics analysis, we uncovered and validated that CALU was associated with TME remodeling, gene mutation and ferroptosis. Further study of CALU is beneficial for precision medicine of BLCA and may provide reliable targets for improving the immunotherapeutic response of BLCA.

## Methods and Materials

### Raw Data Acquisition

The gene expression quantification data for transcriptome profiling included 408 BLCA patients, and the corresponding clinical data (**Table 1**) were downloaded from the TCGA database (https://portal.gdc.cancer.gov/) in which the method of acquisition and application complied with the guidelines and policies.

**Table 1:** Clinical characteristics between CALU^high^ and CALU^low^ groups of TCGA BLCA cohort.

Characteristics	CALU High	CALU Low	P_value
**Alive**	94 (46.0%)	135 (66.2%)	
**Dead**	110 (54.0%)	69 (33.8%)	p<0.001
**FEMALE**	55 (27.0%)	52 (25.5%)	
**MALE**	149 (73.0%)	152 (74.5%)	0.822
**T1**	5 (2.5%)	6 (3.0%)	
**T2**	79 (39.1%)	112 (56.0%)	
**T3**	94 (46.5%)	63 (31.5%)	
**T4**	24 (11.9%)	19 (9.5%)	0.005
**N0**	107 (57.8%)	130 (71.8%)	
**N1**	30 (16.2%)	16 (8.8%)	
**N2**	41 (22.2%)	34 (18.8%)	
**N3**	7 (3.8%)	1 (0.6%)	0.008
**M0**	81 (91%)	115 (97.5%)	
**M1**	8 (9.0%)	3 (2.5%)	0.059
**Stage I**	1 (0.5%)	1 (0.5%)	
**Stage II**	46 (22.7%)	84 (41.4%)	
**Stage III**	76 (37.4%)	64 (31.5%)	
**Stage IV**	80 (39.4%)	54 (26.6%)	p<0.001
**High Grade**	202 (99.5%)	182 (90.0%)	
**Low Grade**	1 (0.5%)	20 (10.0%)	p<0.001

The chi-square and Fisher exact test indicated significant correlation between CALU and survival status(p<0.001), grade (p<0.001), stage (p<0.001), T (p=0.005) and N (p=0.008) classification in BLCA.

### Survival Analysis

The Kaplan-Meier (KM) survival analysis was used to compare the survival difference. The Log-rank test and univariate Cox regression generated p-value and hazard ratio (HR) with 95% confidence interval (CI). Figures were plotted by the “ggrisk,” “survival,” and “survminer” packages of R language version v4.0.3

### Independent Risk Analysis and Nomogram Construction

Univariate and multivariate cox regression analysis were performed to identify the independent risk factors for BLCA prognosis. The P-values were shown in the forest plot. HR and 95% CI of each variable were calculated using the ‘forest plot’ R package. A nomogram was developed based on multivariate Cox proportional hazards analysis results to predict the 1,3, and 5-year overall survival (OS). The nomogram provided a visualized representation of the variables, which can be used to calculate the mortality risk for an individual patient by the points associated with each risk factor through the ‘rms’ R package. C-index and a calibration plot were used to assess the accuracy of the nomogram.

### Differentially Expressed Genes (DEGs) Acquisition and Functional Enrichment Analysis

DEGs between CALU^high^ and CALU^Low^ groups were analyzed by Limma package of R software. The adjusted P-value was analyzed to correct for false-positive results. “Adjusted P < 0.05 and |Log_2_ (Fold Change)| >1” were defined as the thresholds for DEGs screening. Gene Ontology (GO), including molecular function (MF), biological pathways (BP), and cellular components (CC), was used for annotating genes with functions. Kyoto Encyclopedia of Genes and Genomes (KEGG) Enrichment Analysis was used to obtain an analytical study of gene functions and associated high-level genome functional information. ClusterProfiler package of R was employed to analyze the GO function of potential targets and enrich the KEGG pathway. Gene sets enrichment analysis (GSEA) was performed using the Broad Institute’s GSEA program (http://www.broadinstitute.org/gsea/index.jsp). The Hallmark v7.2, c2 Kegg, and c5 Go (BP, CC, MF) gene sets were used for GSEA analysis.

### Gene Mutation Analysis

Somatic mutation data was downloaded from the TCGA database and visualized by the “maftools” package of R language. Mutation information of each gene was shown in the waterfall plot. The various mutation types were annotated with different colors at the top right of the waterfall plot.

### Estimation of the TME

To make reliable estimations of TIICs, we utilized the ‘immunedeconv,’ an R package that integrated six prevalent algorithms, including TIMER, xCell, MCP-counter, CIBERSORT, EPIC and quanTIseq (18). In this article, we displayed the estimation results of TIMER (19) and MCP-counter (20), which included both the immune and stromal components. R package ‘estimate’ was conducted to assess the TME components and tumor purity. Scores of stromal and immune components were then obtained. The ESTIMATE score was the sum of these two and was negatively correlated with tumor purity.

### ICB Treatment Reactiveness Prediction

Immune cell abundance identifier (Immune cell AI, http://bioinfo.life.hust.edu.cn/ImmuCellAI) was used to predict the patients’ responsiveness to ICB therapy on the immune cell infiltration levels.

### scRNA Sequencing Datasets Acquisition

The scRNA sequencing datasets were collected from the TISCH database (http://tisch.comp-genomics.org/home/) (21), which provided detailed cell-type annotation at the single-cell level, enabling the exploration of TME across different cancer types.

### Real-Time Quantitative PCR

Samples were collected from post-operative tissues of 32 BLCA patients. According to the manufacturer’s instructions, triazole (Invitrogen) was used for extracting total RNA from all clinical samples. The quantitative polymerase chain reaction (qPCR), using the SYBR-Green method (TaKaRa), was performed on an ABI 7500 real-time PCR system (Applied Biosystems). The relative expression level of CALU was calculated by the 2−ΔΔCt method after normalizing to β-actin level. Primer sequences of CALU were listed as followed:

CALU Forward (5’-3’) TGGATTTACGAGGATGTAGAGC

Reverse (5’-3’) TTTTAAACCTCCGCTCATCTCT

β-actin Forward (5’-3’) AAACGTGCTGCTGACCGAG

Reverse(5’-3’) TAGCACAGCCTGGATAGCAAC

Gene expression was made average from three individual tests, representing the expression level of CALU for each patient.

### IHC Analysis and IHC Score

Gene expression was detected using the BenchMark GX automatic multifunctional immunohistochemical staining system (Roche, Switzerland) with OptiView DAB Detection Kit (Ventana, USA) according to the manufacturer’s instructions. The primary antibodies (**Table 2**) were visualized by a horseradish peroxidase-labeled secondary antibody. Hematoxylin was used for counterstaining and Bluing Reagent for post counterstaining. Two pathologists (Jiang Xiang & Cao Jin) evaluated the immunohistochemical results without acknowledging the patient’s information. IHC score was calculated according to the staining intensity and the proportion of positive stromal cells. The standard was as followed: [IHC score 1], weak staining in <50% or moderate staining in <20% of stromal cells; [IHC score 2], weak staining in ≥50%, moderate staining in 20-50% or intense staining in <20%; [IHC score 3], moderate staining in ≥50% or intense staining in ≥20%. Cases with scores 2 or 3 were regarded as positive for each protein expression (22).

**Table 2:** Primary antibodies used in IHC analysis.

Primary antibody	Description	Role of gene	Manufacturer		Catalog	Dilution
ACTA2	Actin Alpha 2, smooth muscle	a marker of myCAFs		Abcam	ab7817	1:100
CD206	Mannose receptor C-type 1	a marker of Macrophages M2		Abcam	ab252921	1:4000
CD8	CD8a molecule	a marker of CD8 T cells		Abcam	ab237709	1:100
PDGFRA	Platelet derived growth factor receptor alpha	a marker of iCAFs		Abcam	ab134123	1:500
CALU	Calumenin	an EMT related gene		Abcam	ab137019	1:250

### Statistics Analysis

The association between CALU and clinical variables was analyzed using Pearson chi-square test or Fisher’s exact test. The KM survival analysis with log-rank test was used to compare the survival difference among groups. Univariate and multivariate cox regression analysis were applied for screening the independent risk factors for BLCA prognosis. The Wilcoxon test examined the differences between variables of two groups. Kruskal Wallis test analyzed statistical significance for variables of more than two groups. Fisher exact test was used to identify the correlation of CALU with ICB responsiveness, CD8+ T cells and macrophages. Two sides P-value <0.05 was considered significant. R language v4.0.3 was used for all statistical analyses.

## Results

### CALU Was Correlated With the OS and Progression-Free Survival (PFS) of BLCA Patients

**Figure 1:**
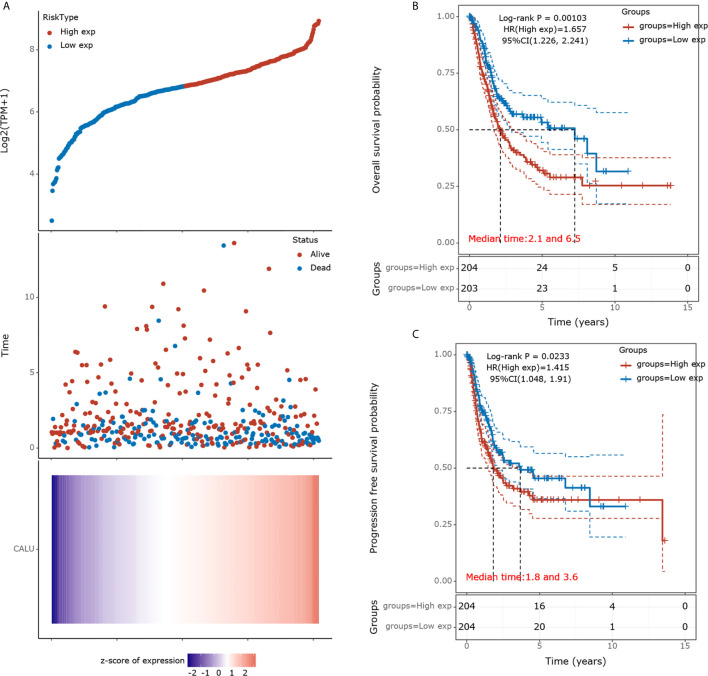
Prognostic analysis of CALU in the TCGA BLCA cohort. **(A)** A scatterplot of CALU expression from low to high was placed in the upper portion. The middle represents the scatter plot distribution of survival time and survival status corresponding to different patients’ CALU expression. The heatmap of CALU expression in all patients was shown at the bottom. **(B)** Kaplan-Meier survival analysis validated CALU as an adverse factor for the OS in BLCA (p=0.001, HR=1.657). **(C)** CALU also played a significant role in affecting the PFS of BLCA patients (p=0.023, HR=1.415).

We divided the 408 BLCA patients into high and low CALU expression groups according to their medium CALU expression level. The survival status of the patient in each group was shown in **Figure 1A**. The KM survival analysis showed a significant difference in OS between CALU^high^ and CALU^Low^ groups, with lower OS in the CALU^high^ group (p=0.001) (**Figure 1B**). Similarly, PFS was significantly lower in the CALU^high^ group than in the CALU^Low^ group, with a P-value of 0.023 (**Figure 1C**).

### CALU Was Closely Associated With the Prognosis of BLCA

**Figure 2:**
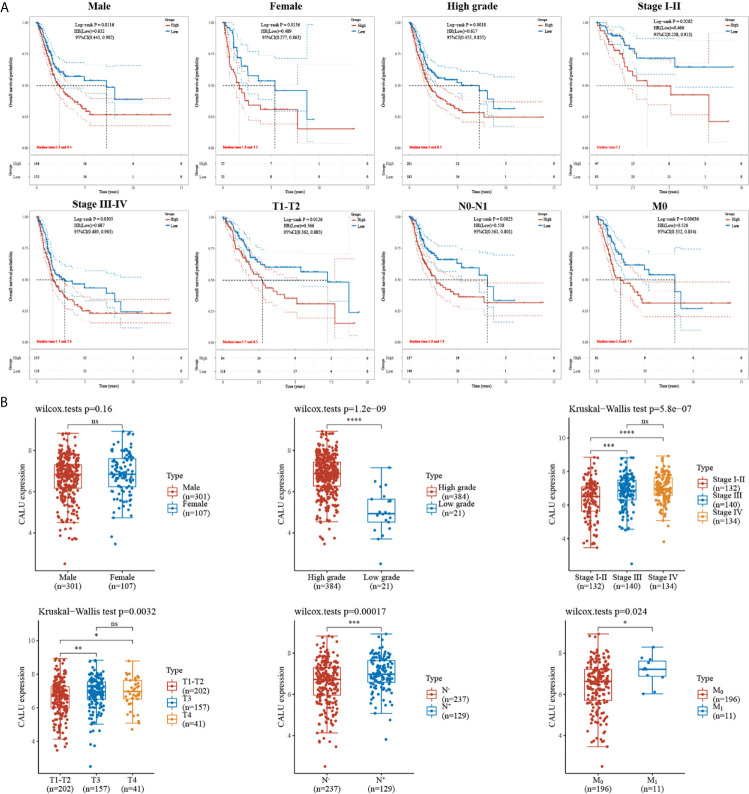
Correlation of CALU with subgroup survival and clinical characteristics. **(A)** the expression level of CALU altered the OS of BLCA patients in multiple subgroups, including male (p=0.012), female (p=0.014), high grade (p=0.002), Stage I-II (p=0.026), Stage III-IV (p=0.031), T1-T2 (p=0.013), N0-N1 (p=0.002) and M0 (p=0.006). **(B)** Tumor tissue with higher grade (p<0.001), stage(p<0.001), T (p=0.003), N (p<0.001),M (p=0.024) classification expressed higher CALU. ****p<0.0001, ***p<0.001, **p<0.01, *p<0.05. NS, not significant.

Subsequently, we subjected these 408 BLCA patients into subgroups. By comparing the survival of patients with high and low CALU expression between different subgroups, we found a strong association between CALU and patients’ OS in multiple subgroups. Specifically, we observed shorter OS in CALU^high^ patients in subgroups including the male (p=0.012), female (p=0.014), high grade (p=0.002), Stage I-II (p=0.026), Stage III-IV (p=0.031), T_1_-T_2_ (p=0.013), N_0_-N_1_ (p=0.002) and M_0_ (p=0.006) (**Figure 2A**). These results further suggested that CALU level was an adverse factor for patients’ OS in BLCA. We also analyzed CALU levels in BLCA patients with different clinical features. Our results indicated that CALU expression increased significantly with increasing BLCA grade, progression in stage and TNM classification (**Figure 2B**).

### Validation of CALU as an Independent Risk Factor for Survival of BLCA and Construction of a Prognostic Nomogram Incorporating CALU With Clinical Features

**Figure 3:**
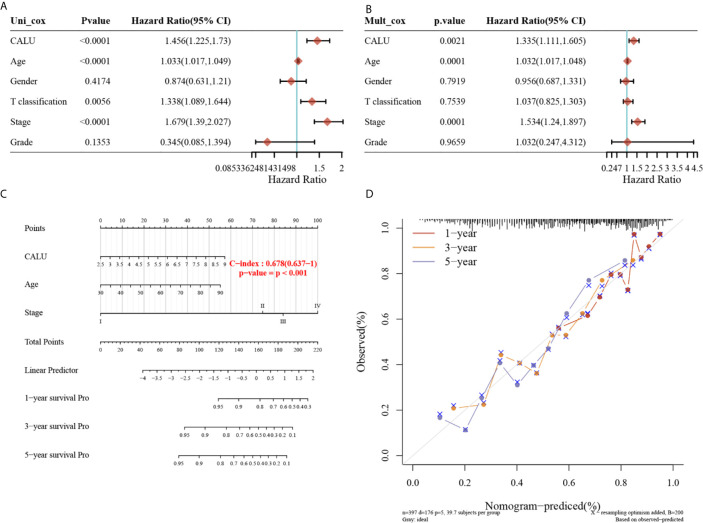
Identification of CALU as an independent risk factor of BLCA and construction of a prognostic nomogram including CALU level. **(A, B)** The univariate (p<0.001) and multivariate (p=0.002) Cox regression analysis confirmed CALU as an independent risk factor for BLCA. **(C)** A nomogram composed of CALU level, age and stage well indicated the OS of BLCA patients based on the nomogram scores, with a c-index of 0.678. **(D)** The calibration curve confirmed a pleasant accuracy of the nomogram in predicting 1,3 and 5 years’ OS.

To further investigate whether CALU could be an independent risk factor for BLCA prognosis, we performed univariate (**Figure 3A**) and multivariate (**Figure 3B**) Cox regression to screen independent prognostic factors for BLCA. The univariate Cox regression analysis revealed that CALU (p<0.0001, HR=1.456, 95%CI:1.225-1.73), age (p<0.0001, HR=1.033, 95%CI:1.017-1.049), T classification (p=0.0056, HR=1.338, 95%CI:1.089-1.644), and stage (p<0.0001, HR=1.679, 95%CI:1.39-2.027) significantly affected the OS of BLCA patients. Subsequent multivariate cox regression analysis suggested that CALU (p=0.0021, HR=1.335, 95%CI:1.111-1.605), age (p=0.0001, HR=1.032, 95%CI:1.017-1.048) and stage (p=0.0001, HR=1.534, 95%CI:1.24-1.897) could be used as independent risk factors for BLCA prognosis. We constructed a prognostic nomogram using these three factors obtained from the multivariate regression, and the C-index of the nomogram was 0.678 (**Figure 3C**). Using the calibration curve, we can see that the prediction model has good accuracy in predicting patients’ survival risk at 1, 3 and 5 years (**Figure 3D**).

### CALU May Be Involved in TME Remodeling in BLCA

**Figure 4:**
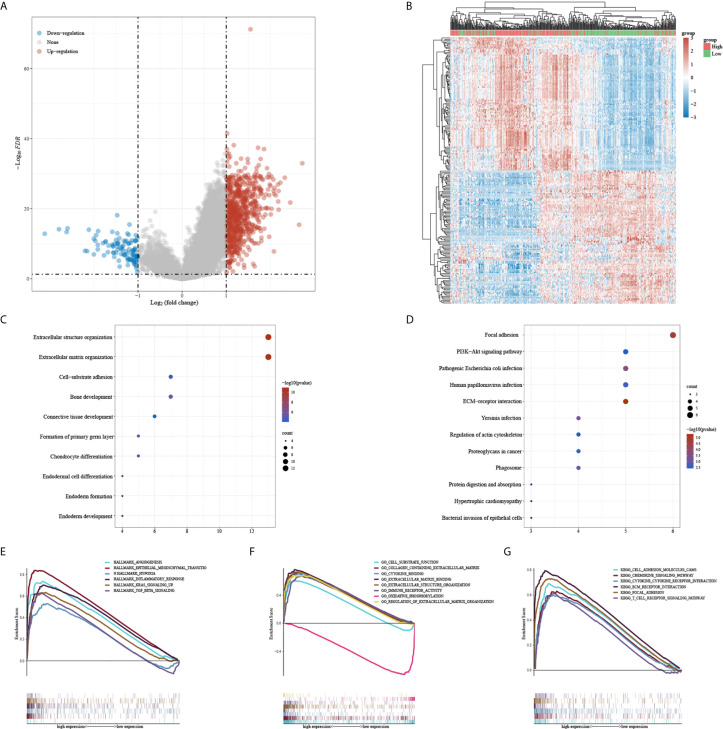
DEGs and functional enrichment analysis between CALU^high^ and CALU^low^ groups. **(A, B)** the DEGs between CALU^high^ and CALU^low^ groups were shown in the heatmap and volcano plot. **(C, D)** GO and KEGG enrichment analysis confirmed that patients with higher CALU expression owned higher extracellular matrix remodeling activities **(E–G)** The GSEA suggested that the CALU high expression group had higher EMT, hypoxia, cytokine receptor and T cell receptor activities.

A total of 751 upregulated genes and 104 down-regulated genes were screened between CALU^high^ and CALU^low^ groups (**Figures 4A, B**). The GO and KEGG enrichment analysis indicated that CALU might be involved in extracellular structure organization, extracellular matrix organization and focal adhesion (**Figures 4C, D**). We further analyzed CALU’s functions by GSEA, and the results suggested that CALU level was positively related to the activities of TME related processes, including EMT, hypoxia, inflammatory response and TGF-β signaling pathways in Hallmark gene sets. While in GO and KEGG gene sets, CALU level was positively associated with enhanced activity of stromal(Extracellular matrix binding, Extracellular structure organization and adhesion molecules cams) and immune-related processes and pathways (immune receptor response and T cell receptor signaling pathway) (**Figures 4E–G**).

### CALU Is Involved in the Regulation of Both the Stromal and Immune Components of BLCA

**Figure 5:**
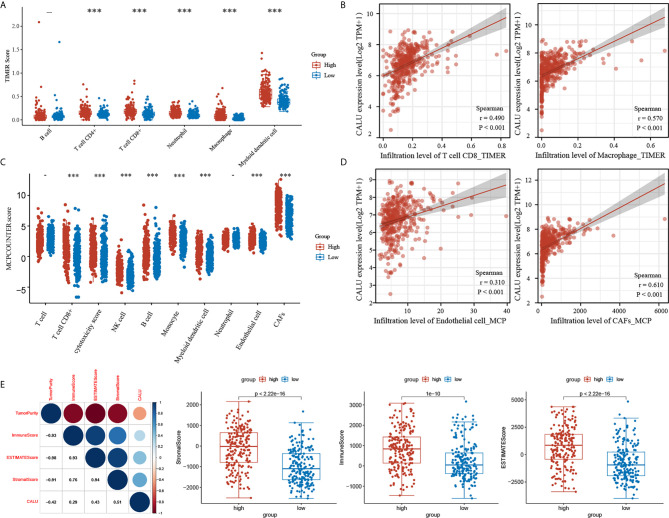
The dual regulatory function of CALU on TME. **(A–D)** The TIMER and MCP-COUNTER algorithms indicated the significant correlation of CALU with CD8 T cells (p<0.001, R=0.490), macrophages(p<0.001, R=0.570), endothelial cells (p<0.001, R=0.310) and CAFs (p<0.001, R=0.610). **(E)** CALU expression level positively correlated with the stromal (p<0.001, R=0.510), immune (p<0.001, R=0.290) and ESTIMATE scores (p<0.001, R=0.430) and negatively associated with tumor purity (p<0.001, R=-0.420). ***p<0.001.

Based on the results of gene enrichment analysis, we have identified that CALU may regulate various tumor microenvironment components. In this regard, we conducted a further analysis by algorithms including TIMER, MCPCOUNTER and ESTIMATE. By the TIMER algorithm, we mainly focused on the correlation between CALU and immune components. The results showed that the level of TIICs such as CD8+ T cells (p<0.001) and macrophages (p<0.001) were significantly higher in the CALU^high^ group than the CALU^low^ group (**Figure 5A**), and there was a significant positive correlation of CALU with CD8+ T cells (R=0.490, p<0.001) and macrophages (R=0.570, p<0.001) (**Figure 5B**). Through the MCP-COUNTER algorithm, we revealed that endothelial cells (p<0.001)and CAFs (p<0.001) were significantly higher in the CALU^high^ group than the CALU^low^ group (**Figure 5C**), and there was a highly positive correlation between CALU and endothelial cells (R=0.310, p<0.001) and CAFs (R=0.610, p<0.001), especially CAFs (**Figure 5D**). The stromal and immune scores were calculated by the ESTIMATE algorithm and summed to obtain the ESTIMATE score. After that, we found that CALU was positively correlated with all three score forms and negatively correlated with tumor purity. Among the three scores, CALU had the highest correlation with the stromal score (R=0.510), suggesting that CALU is closer with the stromal component than with the immune component and laterally suggesting that it may be involved in the immune regulation of BLCA by acting on the stromal components (**Figure 5E**).

### CALU Is Strongly Associated With Multiple Immune Checkpoint-Related Genes (ICRGs) and May Affect Patient Responsiveness to Immunotherapy

**Figure 6:**
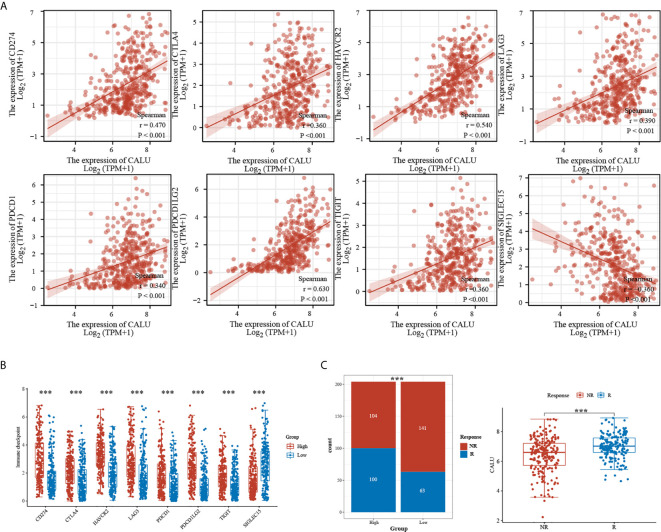
Significant correlation of CALU with multiple ICRGs. **(A, B)** CALU was positively correlated with CD274 (p<0.001, R=0.470), CTLA4 (p<0.001, R=0.360), HAVCR2 (p<0.001, R=0.540), LAG3 (p<0.001, R=0.390), PDCD1 (p<0.001, R=0.340), PDCD1LG2 (p<0.001, R=0.630), and TIGIT (p<0.001, R=0.360), while negatively correlated with SIGLEC15 (p<0.001, R=-0.360). **(C)** ICB therapy responsiveness predicted by Immunecell AI indicated a significantly higher response rate in CALU^high^ patients than in CALU^low^ patients(p<0.001). The CALU expression level in ICB responders was higher than that in non-responders (p<0.001). ***p<0.001.

We further analyzed the correlation between CALU and multiple ICRGs and found that CALU had significant positive correlations with CD274 (PD-L1), CTLA4, LAG3, PDCD1 (PD1), TIGIT, and PDCD1LG2 (PD-L2) (**Figure 6A**). Meanwhile, these ICRGs’ expression levels were significantly higher in the CALU^high^ group than the CALU^low^ group (**Figure 6B**). Since PD-L1 was critical to patient’s ICB responsiveness and there was a significant positive correlation between PD-L1 and CALU, whether the expression level of CALU was also associated with ICB responsiveness, to this end, we predicted the ICB responsiveness in the CALU^high^ and CALU^low^ groups by using the Immunecell AI database. The results revealed more patients responding to immunotherapy in the CALU^high^ group than the CALU^low^ group (p<0.001). Also, by comparing the expression levels of CALU in responders and non-responders, we found that the expression levels of CALU were higher in responders (p<0.001) (**Figure 6C**).

### CALU Was Correlated With TP53 Mutation and Various Ferroptosis Related Genes

**Figure 7:**
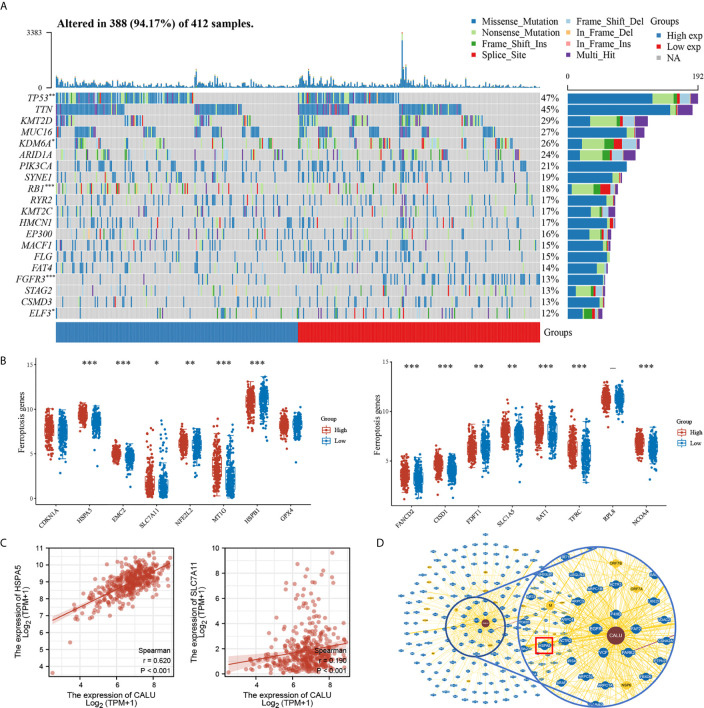
Association of CALU with gene mutations and ferroptosis in BLCA. **(A)** Significant differences in mutation frequency of TP53, RB1, FGFR3 between CALU^high^ and CALU^low^ groups, with higher TP53(p<0.01), RB1(p<0.001) and lower FGFR3 (p<0.001), ELF3 (p<0.05) mutation rates in CALU^high^ groups. **(B, C)** CALU correlates with various of ferroptosis-related genes, including HSPA5 (p<0.001, R=0.620)and SLC7A11 (p<0.001, R=0.190). **(D)** The protein-protein interaction network from the BioGRID database confirmed the interaction between CALU and HSPA5. ***p<0.001, **p<0.01, *p<0.05.

**Figure 8:**
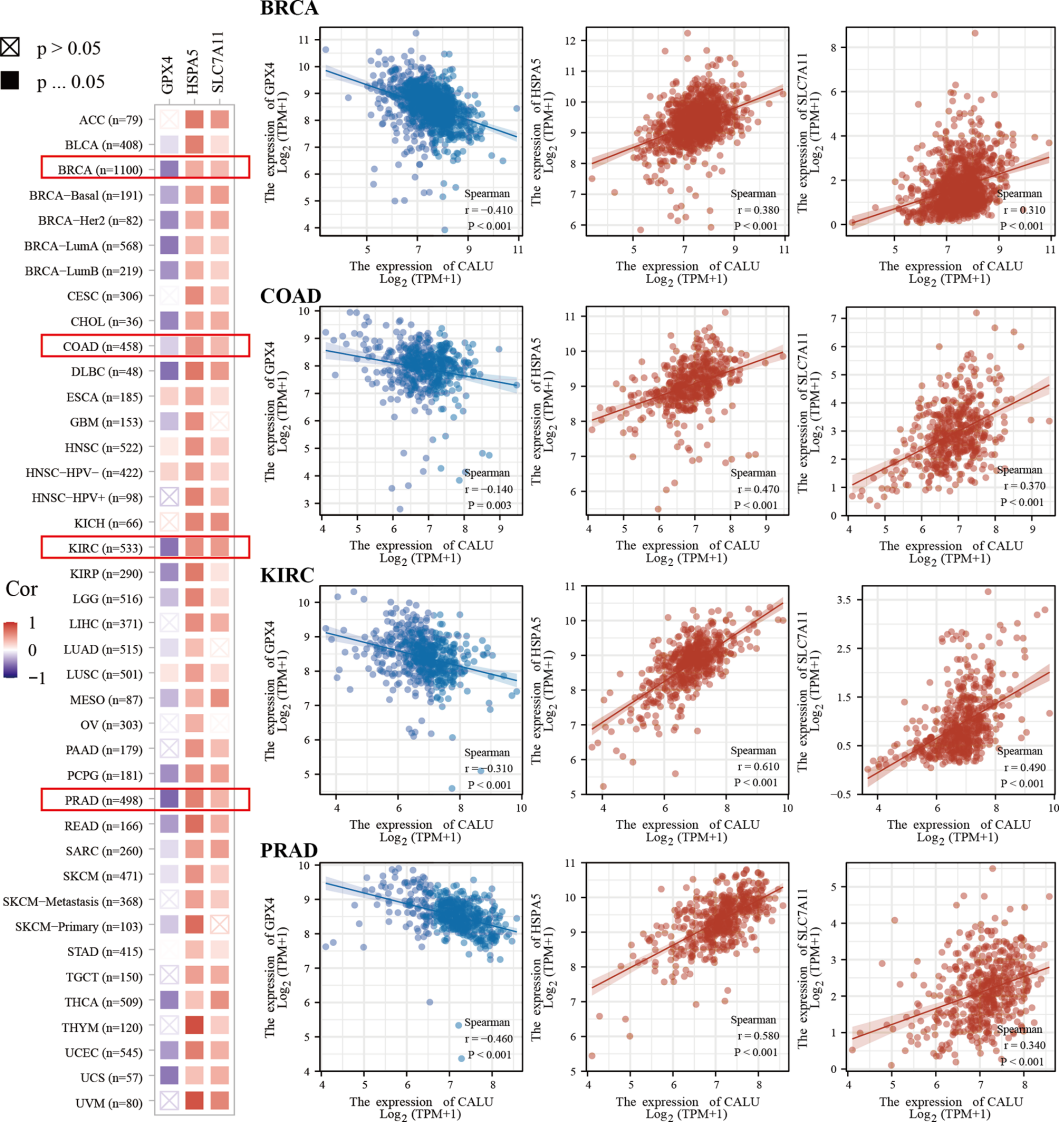
Pan-cancer analysis of the correlation of CALU with GPX4, HSPA5 and SLC7A11. CALU was significantly associated with HSPA5, SLC7A11 and GPX4 in tumors where ferroptosis was commonly observed, including breast, kidney, colon and prostate cancers.

We found higher frequencies of TP53 and RB1 mutations and lower FGFR3 and ELF3 mutations in the CALU^high^ group through somatic mutation analysis (**Figure 7A**). Mutations in these genes were closely associated with the development of BLCA (23, 24). Among them, TP53 was found to have an association with ferroptosis in recent years. For this reason, we further explored the correlation between CALU and ferroptosis. We found that CALU was positively correlated with various ferroptosis-related genes through intergroup comparison and the Spearman correlation analysis, including HSPA5 and SLC7A11 (**Figure 7B**). The correlation between CALU and HSPA5 was the most significant. As a downstream gene of TP53, SLC7A11, which could inhibit ferroptosis, was also associated with CALU (**Figure 7C**). Through the protein-protein interaction network analysis from the BioGRID database, we found that the binding of CALU to HSPA5 protein was confirmed in several experimental results (**Figure 7D**). Though GPX4, a key gene related to ferroptosis, was not correlated to CALU in BLCA. The association of CALU with GPX4, HSPA5 and SLC7A11 in the pan-cancer data revealed a significant relationship between CALU and ferroptosis among various tumors, especially the breast, prostate and kidney cancers where ferroptosis was more commonly observed (**Figure 8**).

### Validation of the Roles of CALU by scRNA Sequencing and Clinical Bladder Cancer Sections

**Figure 9:**
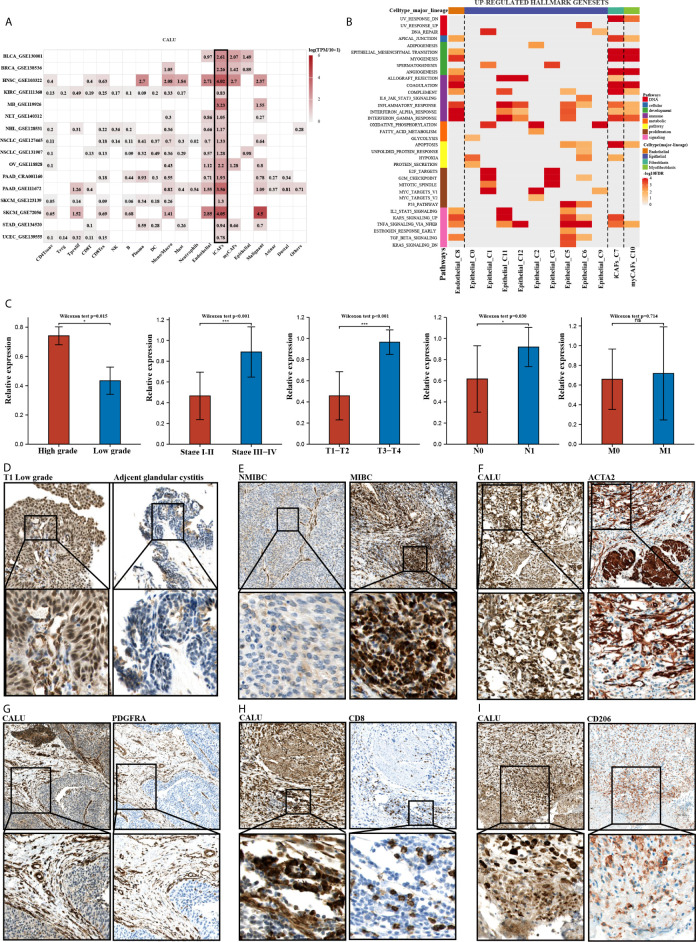
Clinical validation of the roles of CALU. **(A, B)** CALU was confirmed to be expressed by CAFs, especially iCAFs, by scRNA sequencing. The enriched hallmark gene sets by iCAFs were highly corresponded with the genesets which TCGA CALU^high^ group enriched. **(C)** PCR analysis confirmed the differential expression of CALU in tumors with different grades (p<0.05), stages (p<0.001), and T (p<0.001), N (p<0.05) classifications. **(D, E)** Immunohistochemical analysis indicated that CALU levels were higher in BLCA than in GC, while CALU levels were higher in MIBC than in NMIBC. **(F, G)** CALU co-expressed with ACTA2 **(F)** and PDGFRA **(G)** by stromal components **(H, I)** Co-expressions of CALU, CD206 and CD8 were observed in MIBC samples. ***p<0.001, *p<0.05. NS, not significant.

To further validate the correlation between CALU and CAFs, we analyzed the pan-cancer single-cell sequencing datasets from the TISCH database. These datasets classified the stromal cellular components into four categories: “epithelial cells,” “endothelial cells,” “fibroblasts,” and “myofibroblasts.” We further analyzed the annotated “fibroblasts” and “myofibroblasts” in the database for relevant markers. The results indicated that the “fibroblasts” annotated here expressed high expression of PDGFRA, CXCL12, CFD, DPT, and CXCL1, markers which are consistent with the characteristics of inflammatory fibroblasts (iCAFs) (**Table 3**). The results of the TISCH database were in line with the classification of CAFs into iCAFs and myCAFs in previous literature (25). The pan-cancer datasets consistently found that CAFs, especially iCAFs, highly expressed CALU (**Figure 9A**). GSEA analysis of single cells revealed that the hallmark gene sets enriched by iCAFs expressed genes were highly consistent with the CALU^high^ group in the TCGA cohort, especially hypoxia and KRAS signaling up, two gene sets that are not enriched in myCAFs (**Figure 9B**).

**Table 3:** Marker gene expression of ‘Fibroblasts’ and ‘myofibroblasts’ of TISCH database.

Marker gene	Fibroblasts			Myofibroblasts			cell type marker
	Log2FC	Percentage(%)	Adjusted P-value	Log2FC	Percentage(%)	Adjusted P-value	
**PDGFRA**	0.77	41	2.58E-268	—	—	—	**Marker gene of iCAFs**
**CXCL12**	0.64	25.7	1.39E-85	—	—	—
**CFD**	2.26	29.9	2.03E-25	—	—	—
**DPT**	1.42	47.9	0.00E+00	—	—	—
**CXCL1**	0.48	17.4	8.31E-10	—	—	—
**ACTA2**	2.03	72.2	8.48E-147	3.61	97.1	6.35E-146	**Marker gene of myCAFs**
**TAGLN**	2.84	85.4	2.78E-195	2.93	97.1	3.71E-127
**MYL9**	2.05	88.2	1.09E-103	2.71	98.6	1.03E-70
**POSTN**	2.77	79.9	0.00E+00	0.56	35.7	2.71E-28
**TPM2**	—	—	—	2.33	91.4	1.12E-82

The annotated ‘Fibroblasts’ expressed high levels of marker genes of inflammatory CAFs, and the ‘myofibroblasts’ expressed genes that indeed expressed by myofibroblasts as reported previously.

Subsequently, we recruited 32 BLCA patients with different TNM classifications and pathological grades to validate the results obtained from bioinformatics analysis (**Table 4**). Tumor samples were collected after clinical surgery by transurethral resection of bladder tumor (TURBT) or radical cystectomy. After PCR analysis, CALU expression levels in patients with higher clinical stages and pathological grades were confirmed higher than those with lower stages and grades (**Figure 9C**). Interestingly, we observed adjacent glandular cystitis (GC), a precancerous lesion of BLCA, and tumor tissue in the same section, and the expression of CALU in tumor cells was significantly higher than that in GC (**Figure 9D**). Meantime, IHC results indicated a significantly higher expression of CALU in MIBC than non-muscle-invasive bladder cancer (NMIBC) (**Figure 9E**). By comparing the expression of two CAFs markers (ACTA2, marker of myCAFs, and PDGFRA, marker of iCAFs) with CALU, we confirmed the co-expression of these three genes by stromal components in adjacent sections (**Figures 9F, G**). Through the IHC score, we tested the correlation of CALU with CD8+ T cells and macrophages M2 (p=0.025) (**Figures 9H, I** and **Table 5**). Although we did not confirm the correlation between CALU and CD8 in our clinical sections, all the above results still highly suggested that CALU affected BLCA prognosis associated with TME remodeling.

**Table 4:** Clinical information of the recruited BLCA patients.

CALU	Stage		Grade		T		N		M	
	Stage I-II	Stage III-IV	High grade	Low grade	T1-T2	T3-T4	N0	N1	M0	M1
**Low**	14	2	10	6	16	0	15	1	15	1
**High**	3	13	14	2	3	13	12	4	14	2
**P**	p<0.001		ns		p<0.001		ns		ns	

CALU level was correlated with Stage (p<0.001) and T classification (p<0.001) in our recruited BLCA patients. ns, not significant.

**Table 5:** Co-expression of genes in BLCA stroma.

Variable	n	CALU(Positive) n(%)	CALU(Negative) n(%)	P-value(Fisher exact test)
**CD206**				
Positive	25	24(96.0%)	1(4.0%)	0.025
Negative	7	4(57.1%)	3(42.9%)	
**CD8**				
Positive	19	17(89.5%)	2(10.5%)	ns
Negative	13	11(84.6%)	2(15.4%)	

Analysis of the IHC score of each patient indicated a significant correlation between CALU and CD206 co-expression in stromal components, suggesting the association of CALU with macrophages. ns, not significant.

## Discussion

BLCA is one of the most commonly diagnosed urological tumors and causes severe cancer-associated mortality worldwide (26). It is generally classified as NMIBC and MIBC (27). In NMIBC, BCG’s intravesical instillation has already been routinely used for over 40 years as immunotherapy to prevent invasive cancer development (28). MIBCs constitute approximately 20% of BLCA incidences but account for the vast majority of cancer‐specific deaths due to poor prognosis (29). Recent advantages in immunotherapies are rapidly updating the treatment options for BLCA. Immune checkpoint inhibitors, including pembrolizumab, atezolizumab, durvalumab, nivolumab and avelumab, were approved by the FDA for the second-line setting metastatic BLCA patients who failed cisplatin-based chemotherapy (30). However, the response rate of ICB was still limited (31)﻿. Accumulating evidence suggested that the TME played an essential role in immunotherapy responsiveness in BLCA (32).

The TME can be divided into stromal and immune components. It is a heterogeneous population of cells consisting of multiple surrounding cells (immune cells and fibroblasts), signaling molecules and the extracellular matrix (33). Interactions of tumor cells with the surrounding microenvironment play a role in tumor invasion capacity, immune invasion and drug resistance (34). The EMT process is an integral part of this interaction (35). From the available evidence, the biological process of EMT involves an intertwined interaction between epithelial cancer cells and the stromal components, conferring a mesenchymal phenotype on tumor cells yielding enhanced invasion and metastasis capacity (14).

CALU was suggested to be a stromal biomarker with prognostic significance in colon cancer (36). It was also identified as a CAFs-related protein, highly expressed in metastasis-positive cases and facilitated lung adenocarcinoma invasiveness (15). However, the role of CALU in BLCA has rarely been reported. In this article, we explored the role of CALU on BLCA and found that CALU correlated with BLCA progression, thus confirming for the first time that CALU is an adverse factor for BLCA prognosis.

Being a hallmark gene, CALU was considered to be involved in the EMT process. In this present article, The results of gene enrichment analysis confirmed that CALU was involved in the EMT process, which may further regulate the extracellular matrix and remodeled the TME. Meanwhile, CALU also participated in the stromal-related pathways, including extracellular matrix remodeling, hypoxia and angiogenesis, and immune-related pathways, including immune receptor activity cytokine binding and T cell receptor pathway. These results indicated the dual regulatory role of CALU to the stromal and immune components of the TME in BLCA.

Studies have linked stromal EMT genes to the immune components in recent years, especially T cell infiltration (14). A positive correlation between T cell infiltration and stromal EMT-related gene expression has been observed in studies of various malignancies, including BLCA (27, 37, 38). However, the relationship between EMT activity and the response to tumor immunotherapy is still controversial. Several studies indicated that tumor patients presenting the higher expression of EMT-related genes should be more likely to benefit from ICB (39), while others correlated EMT-related gene expression with resistance to immunotherapy (40). Further studies of EMT-related genes and cancer immunotherapy may provide potential targets for better immunotherapy responsiveness. In our study, CALU was positively related to the infiltration of CD8+ T cells, which was consistent with the previous studies.

Meanwhile, CALU was positively correlated with multiple ICRGs, such as PD1, PD-L1, PD-L2, CTLA4 and TIGIT. These results suggest that ICB treatment in patients with high CALU expression results in better immunotherapeutic efficacy by sparing more CD8 T cells from immunosuppression caused by PD-L1 expression in tumor cells. Based on this inference, we predicted ICB treatment’s responsiveness among patients with different CALU expression levels by using the Immunecell AI database. Results showed that patients with high CALU expression owned significantly higher responsiveness to ICB therapy than those with low CALU expression, which well confirmed our hypothesis and further suggested that CALU may be involved in regulating CD8 T cell infiltration, modulation of immune checkpoint suppressor molecules and affecting patient responsiveness to ICB therapy. As a protein secreted by CAFs (16), CALU was confirmed to closely related to CAFs, especially iCAFs, in the present study. Our study also revealed a close relationship between CALU and macrophages. Since macrophages also exerted a crucial role in regulating tumor immunity, we believe that stromal EMT-related gene expression could also regulate the tumor immunity through macrophage in addition to CD8+ T cells, but this still needed further verification.

In addition to the regulatory role of the TME, we observed that the expression level of CALU was also closely related to the mutation of genes. The mutations of TP53 and RB1 were significantly higher in the CALU^high^ group than in the CALU^low^ group, while the mutation of FGFR3 was significantly lower in the CALU^high^ group. These three gene mutations played a crucial role in the development of BLCA (23, 24). Meanwhile, recent studies have developed that mutations in TP53 are also closely related to ferroptosis, and SLC7A11, a downstream gene of TP53, is now considered a ferroptosis-related gene (41). To discuss the potential involvement of CALU in regulating ferroptosis, we examined the association between CALU and ferroptosis-related genes, including HSPA5, SLC7A11, MT1G, CISD1, and NCOA4. The results showed a remarkable correlation between CALU and multiple ferroptosis-related genes. As the most significant CALU-associated gene, HSPA5 has been demonstrated to play a vital role in tumor cell’s ferroptosis resistance through interaction with GPX4, a critical gene for ferroptosis (42). In our research, we found CALU indeed interacted with HSPA5 protein through the BioGrid database. A pan-cancer analysis on the association of CALU with HSPA5, GPX4 and SLC7A11 further confirmed the potential involvement of CALU in the regulation of ferroptosis. Although research on ferroptosis was limited in BLCA, a recent study has demonstrated that a biogel system combining photothermal, ferroptotic, and immune therapy through intravesical instillation effectively inhibits BLCA progression (43). These results indicated the potential involvement of CALU in the ferroptosis process and enhanced the application significance of ferroptosis-related therapy in BLCA treatments. Further researches on the potential regulatory role of CALU on ferroptosis may bring new advances in BLCA treatment.

Finally, we validated the functions of CALU on BLCA clinical progression and its modulation of the TME. The findings showed the close association of CALU with iCAFs and that CALU expression levels increased with BLCA progression. Immunohistochemical results from pathological sections of BLCA showed a significant correlation of CALU with macrophage M2. However, we did not find significant correlation between CALU and CD8 T cells in our clinical sections. This might be due to the limited sample volume of our clinical sections. With the results of previous research and our bioinformatics analysis, we still believe CALU could be confirmed to correlate with CD8 T cell infiltration after further researches.

The EMT phenomena are controversial since they could hardly be observed in human bulk tumors. Previous research findings indicated the EMT marker genes were mainly expressed by CAFs, which revealed a complex interaction between stromal components and tumor cells in the EMT process (44). However, suggested as an EMT-related gene, CALU could also be highly expressed in tumor cells, which indicated the crucial roles of CALU in cancer progression other than the function of inducing EMT. In our research, we believe that CALU can be involved in ferroptosis in BLCA from current bioinformatics studies, and we will also conduct in-depth *in vitro* and *in vivo* experiments to validate the exact mechanism of CALU in EMT and ferroptosis in the future.

Despite the insightful findings, limitations still exist in our study. First, relationships between CALU and immune regulation and ICB therapy responsiveness were only verified by bioinformatics analysis. Further verification from *in vitro* and *in vivo* experiments is required for exploring the direct mechanisms of CALU’s immune regulatory function. Second, although a significant correlation was found between CALU and macrophages in BLCA sections, our clinical samples were quite limited. A more extensive validation cohort is still necessary to avoid selection bias. Last, experimental researches should be conducted to explore the possible involvement of CALU in ferroptosis resistance in BLCA.

## Conclusion

In this article, we identified and validated that CALU can be an independent risk factor for BLCA prognosis related to TME remodeling. High expression of CALU significantly increased the responsiveness to ICB treatment, which was associated with higher T-cell infiltration and expression of ICRGs. Given the correlation of CALU with TP53 mutation and multiple ferroptotic genes, our results suggested for the first time that CALU may be involved in ferroptosis regulation through multiple mechanisms. Further in-depth studies of CALU in BLCA will help in the search for targets to increase the responsiveness of tumor immunotherapy and gain a better understanding of the intertwined process of bladder carcinogenesis.

## Data Availability Statement

Publicly available datasets were analyzed in this study. This data can be found here: The datasets used and analyzed during the current study are available from The Cancer Genome Atlas (portal.gdc.cancer.gov) and the TISCH database (http://tisch.comp-genomics.org/home/).

## Ethics Statement

The studies involving human participants were reviewed and approved by Medical Ethics Committee of Shanghai General Hospital. The patients/participants provided their written informed consent to participate in this study.

## Author Contributions

DY and LH designed the study. DY and MW conducted the statistical analysis and draft of the manuscript. JX and CJ did the immunohistochemistry analysis. WY and YJ made the relevant edits to the manuscript and figures. WX and LH revised the manuscript. All authors contributed to the article and approved the submitted version.

## Funding

This study was funded by the National Natural Science Foundation of China (Grant number: 81972371) and Basic Research on Medical and Health Application of Suzhou Municipal Science and Technology Bureau (Grant number: SYSD2020076).

## Conflict of Interest

The authors declare that the research was conducted in the absence of any commercial or financial relationships that could be construed as a potential conflict of interest.
