# Corrigendum: The epithelial to mesenchymal transition related gene calumenin is an adverse prognostic factor of bladder cancer correlated with tumor microenvironment remodeling, gene mutation, and ferroptosis

**DOI:** 10.3389/fonc.2023.1185029

**Published:** 2023-05-11

**Authors:** YiHeng Du, WenHao Miao, Xiang Jiang, Jin Cao, Bo Wang, Yi Wang, Jiang Yu, XiZhi Wang, HaiTao Liu

**Affiliations:** ^1^ Department of Urology, Suzhou Kowloon Hospital, Shanghai Jiaotong University School of Medicine, Suzhou, China; ^2^ Department of Urology, Shanghai General Hospital, Shanghai Jiaotong University School of Medicine, Shanghai, China; ^3^ Department of Pathology, Suzhou Kowloon Hospital, Shanghai Jiaotong University School of Medicine, Suzhou, China

**Keywords:** bladder cancer, calumenin, tumor microenvironment, immunotherapy, gene mutation, ferroptosis

**Error in Author List and Author Contributions**

In the published article, there was an error in the author list, and author WenHao Miao was not listed as co-first author with equal contribution to YiHeng Du. The corrected author list appears below.

YiHeng Du, 1, † WenHao Miao, 2, † Xiang Jiang, 3 Jin Cao, 3 Bo Wang, 1 Yi Wang, 1 Jiang Yu, 1 XiZhi Wang, 1 and HaiTao Liu 2*

The Author Contributions statement has also been updated to reflect this correction. The corrected Author Contributions statement appears below.

DY and MW have equal contributions to the manuscript. DY and LH designed the study. DY and MW conducted the statistical analysis and draft of the manuscript. JX and CJ did the immunohistochemistry analysis. WY and YJ made the relevant edits to the manuscript and figures. WX and LH revised the manuscript. All authors contributed to the article and approved the submitted version.

The authors apologize for this error and state that this does not change the scientific conclusions of the article in any way. The original article has been updated.

